# Plant Growth-Promoting Rhizobacteria Alleviate High Salinity Impact on the Halophyte *Suaeda fruticosa* by Modulating Antioxidant Defense and Soil Biological Activity

**DOI:** 10.3389/fpls.2022.821475

**Published:** 2022-05-26

**Authors:** Rabaa Hidri, Ouissal Metoui-Ben Mahmoud, Walid Zorrig, Henda Mahmoudi, Abderrazak Smaoui, Chedly Abdelly, Rosario Azcon, Ahmed Debez

**Affiliations:** ^1^Laboratory of Extremophile Plants, Centre of Biotechnology of Borj Cedria, Hammam-Lif, Tunisia; ^2^International Center for Biosaline Agriculture, Academic City, United Arab Emirates; ^3^Departamento de Microbiología del Suelo y Sistemas Simbióticos, Estación Experimental del Zaidín, Consejo Superior de Investigaciones Científicas, Granada, Spain

**Keywords:** *Suaeda fruticosa*, PGPR, salt stress, antioxidant enzymes, soil enzymes, mineral nutrition

## Abstract

Plant growth-promoting rhizobacteria (PGPR) are considered as bio-ameliorators that confer better salt resistance to host plants while improving soil biological activity. Despite their importance, data about the likely synergisms between PGPR and halophytes in their native environments are scarce. The objective of this study was to assess the effect of PGPR (*Glutamicibacter* sp. and *Pseudomonas* sp.) inoculation on biomass, nutrient uptake, and antioxidant enzymes of *Suaeda fruticosa*, an obligate halophyte native in salt marshes and arid areas in Tunisia. Besides, the activity of rhizospheric soil enzyme activities upon plant inoculation was determined. Plants were grown in pots filled with soil and irrigated with 600 mM NaCl for 1 month. Inoculation (either with *Pseudomonas* sp. or *Glutamicibacter* sp.) resulted in significantly higher shoot dry weight and less accumulation of Na^+^ and Cl^–^ in shoots of salt-treated plants. *Glutamicibacter* sp. inoculation significantly reduced malondialdehyde (MDA) concentration, while increasing the activity of antioxidant enzymes (superoxide dismutase; catalase; ascorbate peroxidase; and glutathione reductase) by up to 100%. This provides strong arguments in favor of a boosting effect of this strain on *S. fruticosa* challenged with high salinity. *Pseudomonas* sp. inoculation increased shoot K^+^ and Ca^2+^ content and lowered shoot MDA concentration. Regarding the soil biological activity, *Pseudomonas* sp. significantly enhanced the activities of three rhizospheric soil enzymes (urease, ß-glucosidase, and dehydrogenase) as compared to their respective non-inoculated saline treatment. Hence, *Pseudomonas* sp. could have a great potential to be used as bio-inoculants in order to improve plant growth and soil nutrient uptake under salt stress. Indole-3-acetic acid concentration in the soil increased in both bacterial treatments under saline conditions, especially with *Glutamicibacter* sp. (up to +214%). As a whole, *Glutamicibacter* sp. and *Pseudomonas* sp. strains are promising candidates as part of biological solutions aiming at the phytoremediation and reclamation of saline-degraded areas.

## Introduction

Halophytes are plants that are able to thrive and reproduce in habitats where salt concentration exceeds 200 mM NaCl (Flowers and Colmer, 2008). Recently, Kumar et al. (2019) defined halophytes as extremely salt-tolerant plants that naturally grow in saline habitats and have a substantial potential to complete their life cycle in saline environment where no cultivation occurs. Halophytes can be used for reclamation of degraded saline areas, but also as models to better understand adaptive strategies used by these unique species (Rahman et al., 2021). Several studies on halophytes emphasized physiological bases and molecular regulation of salinity tolerance. However, plant salt tolerance is also coupled with complex ecological processes within associated rhizospheric and endospheric microbiomes (Ruppel et al., 2013). Thus, the halophyte microbiome plays a key function in its high tolerance to excessive soil salinity for a better wild crop production. It is thought that the co-evolution of halophytes and plant growth-promoting rhizobacteria (PGPR) has allowed indigenous halophytes to persist in salt habitats (Folli-Pereira et al., 2013; Pan et al., 2020).

Some bacteria from the rhizosphere, known as rhizobacteria, may interact with plants, and affect their growth. Those bacteria are alluded to as PGPR. PGPR have the capacity to help plants to withstand salinity, enabling them to grow and yield better under this osmotic constraint (Egamberdieva et al., 2019; Kumar et al., 2020). On the one hand, PGPR can improve macro- and micronutrient mineral nutrient exchange while also reducing nutritional imbalance caused by increased Na^+^ and Cl^–^ ion influx. Controlling Na^+^ versus K^+^ homeostasis is notably mediated by high-affinity potassium transporters (HKTs). PGPR have been shown to increase Na^+^ exclusion by roots and enhance the activity of HKTs, allowing plants to maintain a greater K^+^/Na^+^ ratio under salt stress (Qin et al., 2016). This is achieved by (i) promoting the development of biofilms on root surfaces and exopolysaccharides (EPS) production (Mukhtar et al., 2019), thus limiting Na^+^ influx into roots, or (ii) *via* tissue-specific downregulation of the expression of HKT1/K^+^ transporter (Gerhardt et al., 2017). Some PGPR also promote plant growth by producing non-volatile compounds, such as the hormones auxin and cytokinin, as well as siderophores, which enhance iron uptake and 1-aminocyclopropane-1-carboxylate (ACC) deaminase, which reduce ethylene levels in the plants (Sagar et al., 2020). In addition, it has been documented that PGPR produce volatile organic compounds which regulate Na^+^ homeostasis pathway in plants. These substances can stimulate induction of HKT1 in shoots and decrease in HKT1 in roots, which facilitate shoot-to-root Na^+^ recirculation (Kaushal and Wani, 2016; Sáenz-Mata et al., 2016).

Plant growth-promoting rhizobacteria can also boost antioxidative systems in plants, such as superoxide dismutase (SOD), catalase (CAT), ascorbate peroxidase (APX), peroxidase (POD), and glutathione reductase (GR), as well as non-enzymatic components like ascorbic acid, cysteine, and glutathione (Islam et al., 2016; Etesami and Maheshwari, 2018).

Halophytes–PGPR collaboration improves not only plant growth but also soil biological activities and other soil status-related properties (Bharti et al., 2015). In saline soils, PGPR play important roles in soil microbial activities as bio-inoculants, hence contributing to the establishment of more favorable conditions for plant growth. PGPR inoculation may improve the physiology of halophyte plants and inner adaptive capacities (Komaresofla et al., 2019). However, there are very few studies in this respect regarding halophytes. *Suaeda fruticosa* (Chenopodiaceae) is a halophilic plant mostly found in Tunisian salt marshes and arid lands (Bankaji et al., 2016). This halophyte displays several applied interests including a source of fodder for camels (Towhidi et al., 2011), edible seed oil of high quality (Weber et al., 2007), and hypoglycemic, hypolipidemic, anti-inflammatory, and anticancer effects of the different plant parts (Benwahhoud et al., 2001; Oueslati et al., 2012).

Besides these useful traits, *S. fruticosa* is an excellent model plant for studying the effect of rhizospheric halobacteria on plant performance under stress conditions, since it is a halophyte that can grow under extreme hypersaline conditions of up to 1,000 mM NaCl. Furthermore, as an include species, *S. fruticosa* cultivation might be useful in the bioremediation and reclamation of salt-affected soils, since it has a high capacity for Na^+^ accumulation (Khan et al., 2009). Based on this available knowledge, the objective of our study was to assess the influence of PGPR inoculation on the growth of *S. fruticosa* plants under salt stress. The following question is addressed: To which extent may PGPR inoculation improve *S. fruticosa* performance under supra-optimal salinity? The main parameters used include biomass accumulation, nutrition uptake, antioxidative enzymatic activities, and rhizospheric soil enzymes.

## Materials and Methods

### Plants and Microorganisms

The halotolerant inocula used were either *Glutamicibacter* sp. or *Pseudomonas* sp. which were isolated from the salt-affected area in *Soliman Sebkha* (North-East Tunisia). Strain identification was performed by the isolation of genomic DNA as described by Pospiech and Neumann (1995) followed by polymerase chain reaction amplification of 16S rRNA as described by Weisburg et al. (1991). Sequence similarity data were obtained using the BLAST analysis tool of NCBI Blast servers. Partial sequence alignment of the 16S rRNA in the two strains showed 100 and 99.87% similarity with *Glutamicibacter* sp. and *Pseudomonas* sp., respectively. They were deposited in GenBank database under accession numbers MK847918 and MK087034, respectively.

Each bacteria isolate was streaked onto Luria Broth (LB) agar plates and incubated at 28°C in obscurity for 24 h. Bacterial cells were then harvested from LB agar plates, transferred into liquid Luria-Bertani liquid medium (LB media), and cultured at 28°C with shaking at 200 rpm to yield 10^7^–10^8^ CFU mL^–1^, as determined by optical density and serial dilutions (Mortensen et al., 1992).

### Plant Material and Culture

Young plants of *S. fruticosa* were propagated by cuttings. Five-cm long stems with a segment carrying leaves were cut from mother plants. These plants were originally collected from the sabkha of El Kelbia Kairouan (35 48′ 57″N, 10 09′ 27′ E, 133 Km to the south from Tunis, semi-arid bioclimatic stage) and later cultivated on sterilized soil (pH 6.65; CE 0.05; assimilable phosphorus 0.24 g/kg; potassium 0.41 g/kg; nitrogen 0.45 g/kg; sodium 0.17 g/kg; chloride 0.05 g/kg; and calcium 0.65 g/kg). Plants were grown in a greenhouse under the following conditions: natural photoperiod, mean temperature (night/day) ranging from 15° to 25°C, and relative humidity of 60–90%. Irrigation (three times a week) was performed using tap water.

After 30 days of the pre-treatment phase, uniform plants were chosen and transferred in pots containing 5 kg sterilized soil (two plants each). Each plant was inoculated with 1 mL of the bacterial culture (10^8^ CFU mL^–1^). After 7 days, inoculated plants were irrigated (three times per week) using tap water at 0 or 600 mM of NaCl. This concentration was chosen considering previous studies which showed that this concentration impacted plant growth but was not lethal (Khan et al., 2000; Hameed et al., 2012). As a whole, there were six treatments (five replicates, i.e., plants each): (1) non-saline non-inoculated plants (control plants, C), (2) saline non-inoculated plants (S), (3) non-saline inoculated plants with *Glutamicibacter* sp. (Glu), (4) non-saline inoculated plants with *Pseudomonas* sp. (Ps), (5) saline inoculated plants with *Glutamicibacter* sp. (S + Glu), and (6) saline inoculated plants with *Pseudomonas* sp. (S + Ps).

After 30 days, plants were harvested and randomly separated in two groups of five plants each. For the first group, leaves for each plant were collected, washed, and divided in half. The first portion was frozen in liquid nitrogen and kept at −80°C for enzyme activity and malondialdehyde (MDA) assays, and the second portion was left for drying for 48 h at 60°C before measuring leaf water content.

Leaf water content (WC) was calculated using the following equation:


WC(%)= [Freshweight(FW)-Dryweight(DW)/Freshweight(FW)]×100.

For the second group, intended for growth analysis and ion assays, plants were divided into shoots and roots, successively rinsed, three times, in cold water, and blotted with a filter paper. The dry weight was measured after 48 h of desiccation at 60°C.

### Relative Electrolyte Leakage

Electrolyte leakage (EL) was determined using an electrical conductivity meter as described by Dionisio-Sese and Tobita (1998). Leaf samples (200 mg) were placed in test tubes containing 10 mL of deionized water. The tubes were incubated in a water bath at 30°C for 2 h, and the initial electrical conductivity of the medium (C0) was measured. Then, the samples were placed in a water bath at 90°C for 30 min, cooled to 25°C, and the final electrical conductivity was measured again (CF). The conductivity of deionized water was also measured and referred to as CW. The percentage of EL was defined as follows:


EL=(C⁢0-CW)/(CF-CW)× 100.

### Lipid Peroxidation

The extent of lipid peroxidation was determined by measuring the MDA content formed through thiobarbituric acid reaction following the method of Hernández and Almansa (2002). Leaf fresh material (200 mg) was homogenized in 2 ml of 0.1% trichloroacetic acid (TCA). Homogenates were centrifuged at 13,000 *g* in a refrigerated centrifuge (HERMLE Z 36 HK) for 20 min. About 0.5 mL of the obtained supernatant was added to 1.5 mL 0.5% (w/v) TBA in 20% (w/v) TCA. The mixture was incubated at 95°C for 30 min, and the reaction was then stopped in a cold-water bath. Samples were centrifuged at 10,000 *g* for 10 min, and the absorbance of the supernatant was read at 532 and 600 nm.

Lipid peroxidation concentration was determined as follows:


MDA⁢(μ⁢m⁢o⁢l⁢g-1)=[((OD⁢ 532-OD⁢ 600)×TV)/(ε×DW)].

$$\text{TV}=\mathrm{Total}\,\mathrm{volume}\,\mathrm{of}\,\mathrm{the}\,\mathrm{extract}\,\left(2\,\text{ml}\right);\,\varepsilon =155\,{\text{mM}}^{-1}\,{\text{cm}}^{-1};\text{DW}=\mathrm{Dry}\,\mathrm{weight}\,\left(g\right).$$

Dryweight(DW)=Freshweight(FW)-[(WC/100)×Freshweight(FW)].

### Protein Determination and Antioxidant Enzyme Activities

Protein concentration was measured using bovine serum albumin as standard, following the method of Bradford (1976). For the extraction of antioxidant enzymes, fresh leaves were homogenized with 1.6 mL of 50 mM KH_2_PO_4_/K_2_HPO_4_ (pH 7.2) containing 1 mM EDTA and 2% PVP and centrifuged at 12,000 *g* for 20 min at 4°C (Aroca et al., 2003).

Total SOD (EC 1.15.1.1) activity was assayed by monitoring the inhibition of the photochemical reduction in nitro blue tetrazolium (NBT) following the method of Burd et al. (2000). One unit (U) of SOD activity was defined as the amount of enzyme required to cause 50% inhibition of NBT photo-reduction at 560 nm.

Catalase (EC1.11.1.6) activity was determined by monitoring the decomposition of H_2_O_2_ at 240 nm for 1 min (Aebi, 1984). The reaction mixture contained 50 mM phosphate buffer (pH 7.0), 10 mM H_2_O_2_, and 100 μl of enzyme extract in a final volume of 2 mL. The activity was quantified using extinction coefficient of 39.6 mM^–1^ cm^–1^.

Ascorbate peroxidase (EC: 1.11.1.11) activity was measured according to the method described by Amako et al. (1994) by monitoring the decrease in absorbance at 290 nm for 1 min. The reaction mixture was comprised of 80 mM potassium phosphate buffer (pH 7.0), 2.5 mM hydrogen peroxide, and 1 M sodium ascorbate in a final volume of 1 ml. The reaction was started with H_2_O_2_, and the activity has been calculated using an extinction coefficient of 2.8 mM^–1^ cm^–1^.

Glutathione reductase (EC: 1.6.4.2) activity was measured by following the change in absorbance at 340 nm due to the oxidation of NADPH (Carlberg and Mannervik, 1985). The reaction mixture (1 mL) containing 50 mM Tris buffer 3 mM MgCl_2_, 1 mM oxidized glutathione, 150 μL enzyme extract, and 0.3 mM NADPH was added and mixed thoroughly to begin the reaction. Enzyme activity was quantified using an extinction coefficient of 6.2 mM^–1^ cm^–1^.

### Soil Enzyme Activities

Soil enzyme activities: dehydrogenase (EC 1.1.1.1), β-glucosidase (EC 3.2.1.21), and urease (EC 3.5.1.5) were measured in fresh rhizosphere soil samples. Dehydrogenase activity was determined by quantifying the rate of reduction in 2-*p*-iodo-nitrophenyl-phenyltetrazolium chloride (INT) to iodo-nitrophenyl formazan (INTF), following the procedures adopted from Garcia et al. (1994). Dehydrogenase activity was measured in 1 *g* of soil, following incubation with 0.2 ml of 0.4% INT in distilled water for 20 h at 22°C in the dark. After incubation, the INTF formed was extracted with 10 ml of methanol with agitation for 1 min and filtered through a Whatman N 5 filter paper. The INTF was read at 490 nm.

β-glucosidase activity was determined by measuring *p*-nitrophenol (PNP) release from *p*-nitrophenyl-β-D−glucopyranoside (PNG, 0.05 M; Tabatabai, 1982). β-glucosidase was measured in 0.5 *g* of soil, following incubation with 0.1 M maleate buffer (pH 6.5) and 0.5 ml of substrate for 60 min at 37°C. The reaction was stopped on ice; TRIS buffer (pH 12) was then added, and the mixture was centrifuged at 3,400 *g* for 8 min. The quantity of PNP formed was measured at 398 nm (Tabatabai and Bremner, 1969).

Urease activity was determined in 0.1 M phosphate buffer at pH 7, and urea (6. 4%) was used as substrates. A soil sample of 0.5 *g* was incubated with two ml of buffer and 0.5 ml of substrate for 90 min at 30°C. Urease activity was determined by the ammonium (NH_4_^+^) released (Nannipieri et al., 1980). The ammonium absorbance was measured at 490 nm.

### Indole-3-Acetic Acid

Six ml of phosphate buffer (pH 7.5) with glucose (1 *g* glucose/100 ml phosphate buffer) and 4 ml of L-tryptophan (l *g* tryptophan/l00 ml H_2_0) were added to 2.0 *g* of soils and incubated at 37°C for 24 h in the dark. Then, 2 ml of 5% TCA solution and 1 ml of 0.5 M CaCl_2_ solution were added (Wöhler, 1997). The soil solution was filtered (Whatman No.2). Three ml of the filtrate was mixed with 2 mL of Salkowski reagent and kept in complete darkness for 30 min, and the absorbance read at 535 nm in each treatment. A standard curve was prepared for Indole-3-acetic acid (IAA; Wöhler, 1997).

### Statistical Analysis

Statistical analysis was performed using SPSS 20.0 statistical program (SPSS Inc., Chicago, IL, United States). The effects of the experimental factors, salt stress, and inoculations as well as the effect of their interactions on plant parameters were assessed by a two-way analysis of variance (ANOVA; Table 1), and means were compared according to Duncan’s test at *P* < 0.05. Principal component analysis (PCA) and correlation analysis was performed using XLSTAT software v. 2014 (Addinsoft, Paris, France).

**TABLE 1:** Two factors ANOVA (bacterial inoculation and saline stress) for all parameters studied of *S. fruticosa* [*F*-values (*P*-values)].

	Glu	Ps	S	S*Glu	S*Ps
SDW	0.234 (0.653)	0.248 (0.639)	20.7 (0.006)*	6.87 (0.028)*	5.17 (0.038)*
RDW	0.452 (0.531)	3.85 (0.107)	17.34 (0.006)*	2.10 (0.175)	0.46 (0.511)
SDW/RDW	0.616 (0.477)	0.091 (0.774)	0.063 (0.813)	7.12 (0.28)	10.87 (0.009)*
S Na^+^	10.2 (0.033)*	4.47 (0.102)	74 (0.001)*	1.23 (0.299)	1.94 (0.201)
S K^+^	0.279 (0.625)	2.47 (0.191)	25.6 (0.004)*	0.258 (0.621)	0.711 (0.419)
S Ca^2+^	29.2 (0.006)*	349.1 (<0.001)*	86.8 (<0.001)*	1.03 (0.334)	19.1 (0.002)*
S Cl^–^	2.46 (0.191)	0.019 (0.897)	73.5 (<0.001)*	0.269 (0.615)	6.63 (0.030)*
R Na^+^	62.5 (0.001)*	19.4 (0.012)*	73 (0.001)*	1.68 (0.299)	12.7 (0.006)*
R K^+^	0.511 (0.514)	1.34 (0.311)	1.31 (0.315)	4.02 (0.080)	1.37 (0.276)
R Ca^2+^	0.136 (0.730)	0.47 (0.530)	4.69 (0.096)	3.82 (0.086)	16 (0.004)*
R Cl^–^	1.77 (0.240)	1.18 (0.325)	21.8 (0.005)*	2.05 (0.183)	0.201 (0.665)
EL	0.197 (0.676)	0.047 (0.840)	17.8 (0.013)*	231 (0.641)	22.9 (0.001)*
MDA	93.9 (<0.001)*	0.217 (0.675)	89.22 (0.001)*	94.2 (<0.001)*	5.22 (0.05)*
CAT	41.7 (0.003)*	80.77 (0.001)*	3.2 (0.148)	219.6 (<0.001)*	25.03 (0.001)*
APX	141.5 (<0.001)*	0.918 (0.392)	115.32 (<0.001)*	0.379 (0.555)	0.013 (0.911)
SOD	0.527 (0.508)	3.011 (0.158)	51.88 (0.002)*	3.25 (0.109)	1.18 (0.308)
GR	0.011 (0.919)	3.22 (0.147)	12.7 (0.023)*	7.87 (0.023)*	2.65 (0.142)
DH	1,977 (<0.001)*	102 (0.001)*	169.3 (<0.001)*	111 (<0.001)*	122 (<0.001)*
URE	20.9 (0.006)*	0.155 (0.709)	43.1 (0.001)*	11.045 (0.009)*	203 (<0.001)*
BGL	63.8 (0.001)*	14.7 (0.018)*	31.29 (0.005)*	89.4 (<0.001)*	54 (<0.001)*
IAA	698.4 (<0.001)*	180 (<0.001)*	2,313 (<0.001)*	14,157 (<0.001)*	372 (<0.001)*

*SDW, shoot dry weight; RDW, root dry weight; SDW/RDW, shoot dry weight/root dry weight ratio; MDA, malondialdehyde; CAT, catalase activity; APX, ascorbate peroxidase activity; SOD, superoxide dismutase activity; GR, glutathione reductase activity; DH, dehydrogenase; URE, urease; BGL, β-glucosidase; IAA, indole- 3-acetic acid; S Na^+^, shoot sodium content; S K^+^, shoot potassium content; S Ca^2+^, shoot calcium content: S Cl^–^, shoot chloride content; R Na^+^, root sodium content; R K^+^, root potassium content; R Ca^2+^, root calcium content; and R Cl^–^, root chloride content.*

*Stands for significant effect.

## Results

### Plant Growth

**FIGURE 1:**
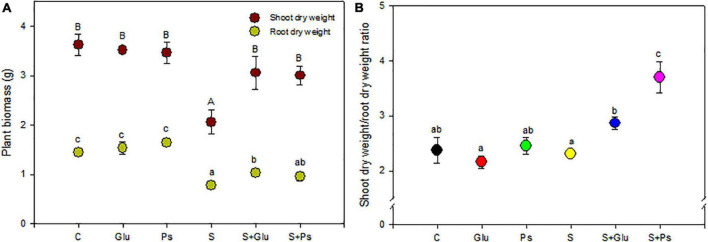
Effect of bacterial inoculation on shoot and root dry weight **(A)** and shoot dry weight/root dry weight ratio **(B)** of *S. fruticosa*, in the absence (0 mM NaCl) and in the presence of NaCl (600 mM NaCl). Values are the means standard deviation of five replicates. Values sharing the different letters indicate significant differences between treatments at the 5% level (Duncan’s).

Salinity significantly affected shoot dry weight (*F* = 20.7; *p* < 0.05) and root (*F* = 17.34; *p* < 0.05) of *S. fruticosa* (Table 1). DW of both shoots and roots of S plants were significantly lower as compared to C plants (Figure 1A). In the presence of NaCl, both bacterial strains significantly mitigated the negative effect of high salinity on plants, compared to non-inoculated plants (whether salt-stressed or not). Indeed, S + Glu and S + Ps treatments increased shoot dry weight, respectively, as compared to S plants (Figure 1A). Inoculated plants challenged with high salinity and especially S + Ps plants showed the highest shoot dry weight/root dry weight ratio compared to non-inoculated plants under both non-saline and saline conditions (Figure 1B).

### Ion Accumulation

**FIGURE 2:**
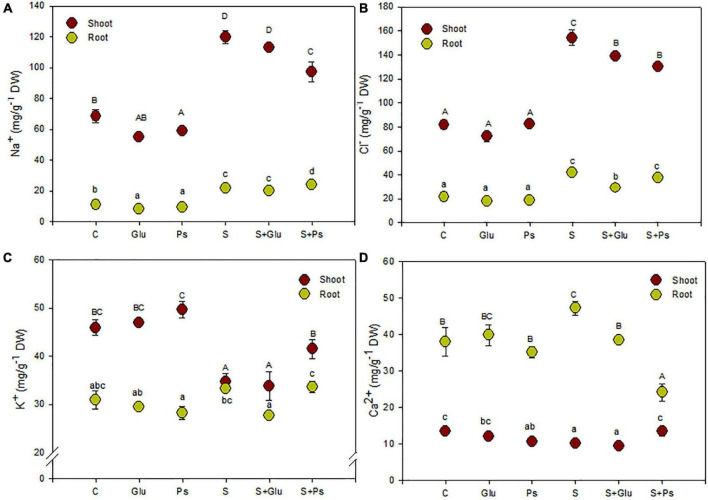
Effect of bacterial inoculation on nutrient accumulation in shoot and root tissues of *S. fruticosa* (mg/g DW): **(A)** sodium, **(B)** chloride, **(C)** potassium, and **(D)** calcium, in the absence (0 mM NaCl) and in the presence of NaCl (600 mM NaCl). Values are the means standard deviation of five replicates. Values sharing the different letters indicate significant differences between treatments at the 5% level (Duncan’s).

Generally, inoculation with *Pseudomonas* sp. and *Glutamicibacter* sp. had no significant effect on plant nutrient uptake as compared to non-inoculated–control plants (C) in salt-free conditions (Figure 2). In contrast, several changes were seen due to bacterial strains inoculation under stress conditions (Figure 2).

In salt-free conditions, inoculation with *Pseudomonas* sp. slightly decreased Na^+^ content in both shoots and roots organs and Ca^2+^ content in roots as compared to non-inoculated–control plants (C; Figures 2A,D). In the case of *Glutamicibacter* sp.-inoculated plants, only root Na^+^ content was decreased compared with control plants (C; Figure 2A).

Salt addition to the irrigation solution increased significantly Na^+^ and Cl^–^ contents in both shoots and roots as compared to control plants (C), this trend being more pronounced in shoots than in roots (Figures 2A,B). Plants exposed to high salinity (S) showed a significant decrease in K^+^ and Ca^2+^ contents in shoot tissues, while they also showed an increase in Ca^2+^ content in roots of stressed plants as compared to C plants (Figures 2C,D).

Under saline conditions, inoculation of the plants with bacterial strains (S + Ps and S + Glu) significantly decreased shoot Cl^–^ content as compared to uninoculated salt-stressed plants (S; Figure 2B). S + Ps treatment led to higher shoot Ca^2+^ and, to a lesser extent, K^+^ contents, whereas root Ca^2+^ content significantly decreased compared to S plants under saline condition (Figures 2C,D).

### Oxidative Stress Biomarkers

**FIGURE 3:**
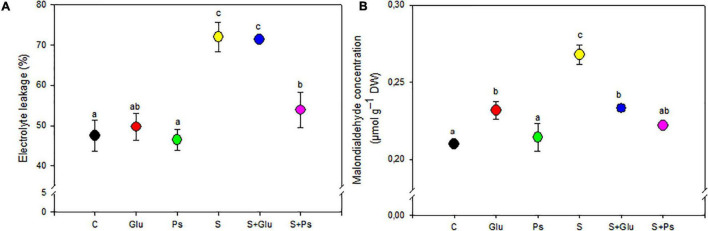
Effect of bacterial inoculation on EL **(A)** and MDA **(B)** content of *S. fruticosa* shoots, in the absence (0 mM NaCl) and in the presence of NaCl (600 mM NaCl). Values are the means standard deviation of five replicates. Values sharing the different letters indicate significant differences between treatments at the 5% level (Duncan’s).

Under non-saline conditions, irrespective of inoculation, there was no significant change in EL and shoot MDA content, except for Glu plants, which showed a significant increase compared to the control (Figures 3A,B). When plants were exposed to salt stress, a significant decrease in values of both EL and MDA was generally observed in PGPR-inoculated (S + Ps and S + Glu) plants compared to non-inoculated stressed (S) plants, except EL in (S + Glu) treatment (Figures 3A,B).

### Antioxidant Enzyme Status

**FIGURE 4:**
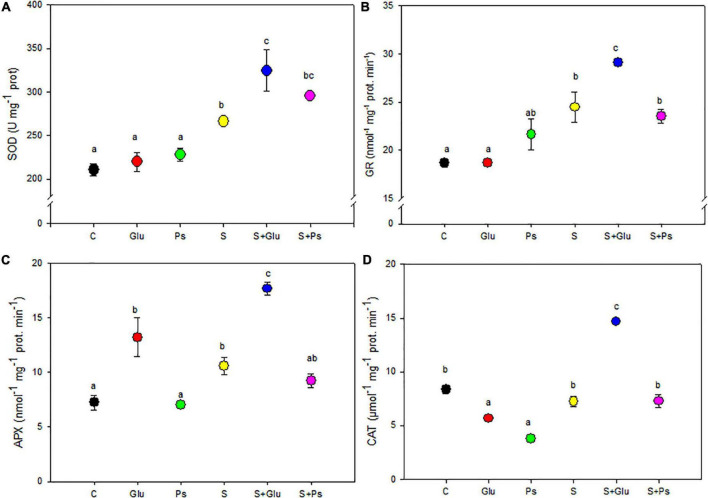
Effect of bacterial inoculation on antioxidant enzyme activities: **(A)** superoxide dismutase (SOD), **(B)** glutathione reductase (GR), **(C)** ascorbate peroxidase (APX), and **(D)** catalase (CAT) in leaves of *S. fruticosa* plants, in the absence (0 mM NaCl) and in the presence of NaCl (600 mM NaCl). Values are the means standard deviation of five replicates. Values sharing the different letters indicate significant differences between treatments at the 5% level (Duncan’s).

Superoxide dismutase and GR activities were comparable in both non-inoculated and inoculated plants under non-saline conditions (Figures 4A,B). Application of salt stress increased activities of both SOD and GR enzymes irrespective of the bacterial treatments, but S + Glu plants showed significantly higher values in comparison with stressed non-inoculated (S) plants (Figures 4A,B). Besides, PGPR-inoculated plants showed significantly lower CAT activity in comparison with control plants under non-saline conditions, whereas a significant increase in APX content was registered in Glu plants (Figure 4C). There was no significant change in CAT activities in salt-stressed (S) as compared to C plants. S + Glu plants showed increased values of CAT and APX activities in comparison with S plants (Figures 4C,D). Overall, the highest values for antioxidant enzyme activities were found salt-stressed plants inoculated with *Glutamicibacter* sp. (Figure 4).

### Soil Enzyme Activities and Indole-3-Acetic Acid Content

**FIGURE 5:**
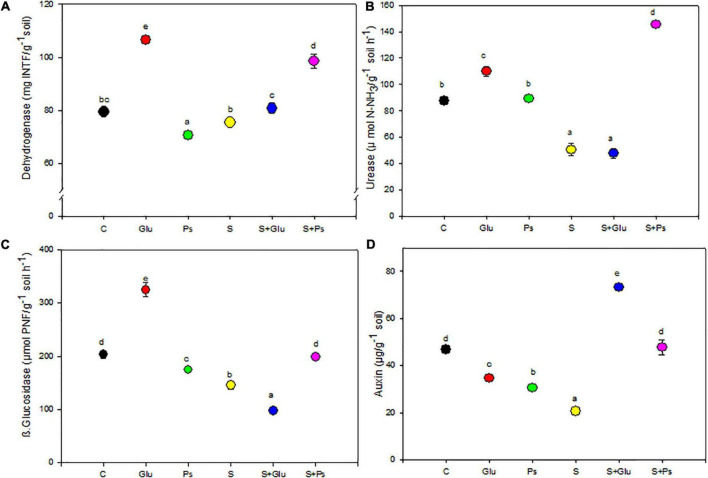
Effect of bacterial inoculation on soil enzymatic activities (mU g^–1^ of dry soil): **(A)** dehydrogenase, **(B)** urease, **(C)** β-glucosidase, and **(D)** IAA production of the rhizospheric soil of *S. fruticosa* plants, in the absence (0 mM NaCl) and in the presence of NaCl (600 mM NaCl). Values are the means standard deviation of five replicates. Values sharing the different letters indicate significant differences between treatments at the 5% level (Duncan’s).

Soil enzyme activities were adversely affected upon irrigation with high salinity, with a significant (*p* < 0.001) reduction in the activities of urease and β-glucosidase as compared to C treatment. In the absence of NaCl, dehydrogenase activity significantly (*p* < 0.001) increased in the rhizosphere of Glu plants while slightly decreasing with *Pseudomonas* sp. compared to the non-inoculated control (C; Figure 5A). Both inoculation treatments (S + Ps and S + Glu) significantly increased dehydrogenase activity in the rhizosphere compared to the non-inoculated salt-stressed (S) plants (Figure 5A). Inoculation with *Glutamicibacter* sp. (Glu) led to a significant (*p* < 0.05) increase in urease activity in salt-free conditions (Figure 5B). Following salt addition, urease activity strongly and significantly (*p* < 0.001) increased in the rhizosphere of S + Ps plants (Figure 5B).

β-glucosidase activity significantly (*p* < 0.05) increased in the rhizosphere of plants inoculated with *Glutamicibacter* sp. in absence of salt but decreased with *Pseudomonas* sp., as compared to the non-inoculated (C) rhizosphere (Figure 5C). Under high salinity, *Pseudomonas* sp. significantly (*p* < 0.001) increased β-glucosidase activity, while *Glutamicibacter* sp. decreased this activity (Figure 5C). Regarding IAA, both bacteria treatments decreased significantly its concentration compared to non-inoculated plants in the absence of NaCl (Figure 5D). Under high salinity, both inoculation treatments (S + Ps and S + Glu) significantly (*p* < 0.001) increased IAA content in the rhizosphere compared to non-inoculated (S) rhizosphere (Figure 5D).

### Correlation Analysis and Principal Component Analysis

**FIGURE 6:**
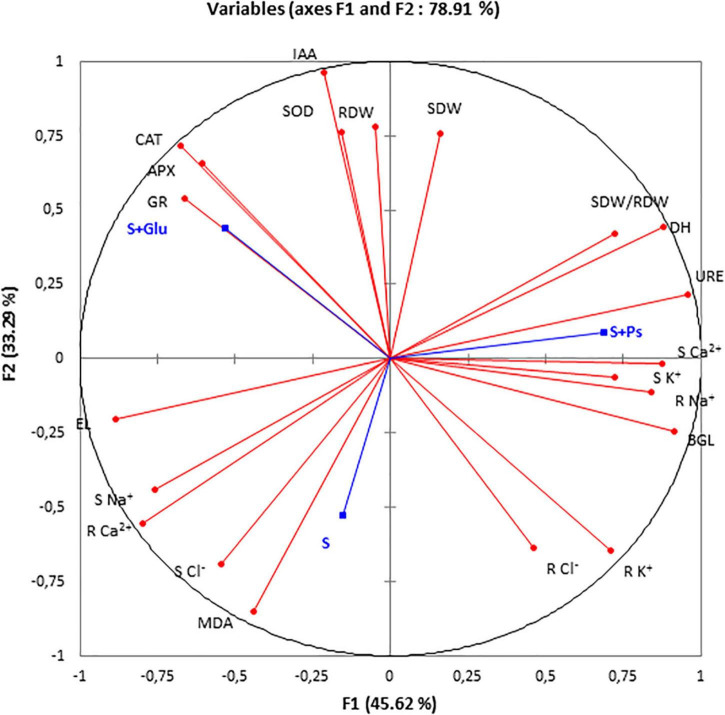
Principal component analysis (PCA). Circles (∙) represent different analysis parameters. Squares (■) represent different treatments (S, S + Ps, and S + Glu). All studied parameters and the different treatments are projected onto the F1–F2 principal factorial plane that explains 78.91% of the variation. SDW, shoot dry weight; RDW, root dry weight; SDW/RDW, shoot dry weight/root dry weight ratio; MDA, malondialdehyde; CAT, catalase activity; APX, ascorbate peroxidase activity; SOD, superoxide dismutase activity; GR, glutathione reductase activity; DH, dehydrogenase; URE, urease; BGL, β-glucosidase; IAA, indole-3-acetic acid; S Na^+^, shoot sodium content; S K^+^, shoot potassium content; S Ca^2+^, shoot calcium content; S Cl^–^, shoot chloride content; R findings Na^+^, root sodium content; R K^+^, root potassium content; R Ca^2+^, Root calcium content; and R Cl^–^, root chloride content.

For a more accurate interpretation of our data, a PCA (Figure 6) and correlation analysis (Table 2) were performed, taking into account all determined parameters characterizing plants and rhizospheric soil under salt stress and bacterial inoculation (S, S + Ps, and S + Glu). Globally, these analyses confirmed the observed positive effect of *Pseudomonas* sp. and *Glutamicibacter* sp. inoculation, enabling *S. fruticosa* to successfully cope with salt stress. High salinity conditions (S) were strongly negatively correlated with plant growth-related traits (shoot and root dry weight, SDW/RDW ratio) and IAA (Figure 6 and Table 2). Besides, S treatment was positively correlated with MDA content, shoot Cl^–^ content, and root Ca^2+^ content. Positive correlations were found under *Pseudomonas* sp. inoculation (S + Ps) for SDW/RDW ratio, shoot K^+^ and Ca^2+^ contents, dehydrogenase, urease, and ß-glycosidase activities. By contrast, this treatment was found to be negatively correlated with shoot Na^+^ content, root Ca^2+^ content, and EL. In S + Glu treatment, positive correlations were observed for CAT, APX, GR, and IAA, while negative correlations were observed for root Na^+^, K^+^, and Cl^–^ contents as well as for β-glucosidase activity. Finally, the results obtained by PCA and correlation analysis showed a perfect match with our trait-by-trait analyses.

**TABLE 2:** Pearson’s correlation matrix analyzing S, S + Glu, and S + Ps treatments and different studied parameters.

Treatments	S	S + Glu	S + Ps	

Parameters				
SDW	−0.81**	0.46	0.40	1
RDW	−0.75*	0.58	0.13	0.9
SDW/RDW	3−0.75*	−0.08	0.83**	0.8
S Na^+^	0.61	0.18	−0.79*	0.7
S K^+^	−0.34	−0.39	0.71*	0.6
S Ca^2 +^	−0.23	−0.53	0.82**	0.5
S Cl^–^	0.74**	−0.19	−0.59	0.4
R Na^+^	−0.15	−0.66*	0.80**	0.3
R K^+^	0.41	−0.92***	0.52	0.2
R Ca^2 +^	0.76*	0.14	−0.90**	0.1
R Cl^–^	0.49	−0.80**	0.37	0
EL	0.40	0.44	−0.87**	−0.1
MDA	0.93***	−0.31	−0.60	−0.2
CAT	−0.49	0.98***	−0.49	−0.3
APX	−0.50	r0.89**	−0.39	−0.4
SOD	−0.67	0.66	0.00	−0.5
GR	−0.31	0.85**	−0.54	−0.6
DH	−0.65	−0.28	0.94***	−0.7
IAA	−0.87	0.85**	0.02	−0.8
URE	−0.47	−0.52	0.99***	−0.9
BGL	−0.03	−0.84**	0.86**	−1

Positive correlations are presented in blue and negative correlations in red. Color intensity is proportional to the correlation coefficient value. Values in bold represent the statistically significant correlations at 0.05 (*), 0.01 (**), and 0.001 levels (***).

*SDW, shoot dry weight; RDW, root dry weight; SDW/RDW, shoot dry weight/root dry weight ratio; MDA, malondialdehyde; CAT, catalase activity; APX, ascorbate peroxidase activity; SOD, superoxide dismutase activity; GR, glutathione reductase activity; DH, dehydrogenase; URE, urease; BGL, β-glucosidase; IAA, indole-3-acetic acid; S Na^+^, shoot sodium content; S K^+^, shoot potassium content; S Ca^2+^, shoot calcium content; S Cl^–^, shoot chloride content; R Na^+^, root sodium content; R K^+^, root potassium content; R Ca^2+^, root calcium content; and R Cl^–^, root chloride content.*

## Discussion

The restoration of a degraded saline areas was most successful using salt-tolerant plant species inoculated with soil microbiota, including PGPR (Qin et al., 2018). Therefore, PGPR are crucial to halophytic plant development, growth, and salt stress tolerance (Komaresofla et al., 2019). However, the ecological function of halophyte symbiotic microbes, including the growth promotion of host and reduced salt stress damage, has been poorly reported.

*Suaeda fruticosa* is a plant native to sabkha ecosystems in the semi-arid bioclimatic stage, where salt levels are typically higher than those of saltwater (Houmani et al., 2015). The optimal salinity for maximum growth of halophytes including *S. fruticosa* is in the range of 200–400 mM NaCl, and its growth is significantly decreased if the soil salinity is outer this range (Khan et al., 2000). Our findings also indicate that high salinity (600 mM NaCl) significantly reduced shoot and root dry weight of *S. fruticosa*. According to Hameed et al. (2012), the growth decline caused by high salinity (600 mM NaCl) may be ascribed to (i) a decreased capacity of water absorption by roots, (ii) a limited ability of the plant to osmotically adjust, and (iii) a toxic ionic effect due to excessive absorption of Na^+^ and Cl^–^, which causes disturbances in nutrient uptake and vital metabolic functions like photosynthesis. Neumann (2011) assumes that growth decrease in response to salt stress might represent an adaptation to boost the odds of surviving long enough to generate seeds.

The present investigation showed that PGPR inoculation significantly increased shoot biomass of *S. fruticosa* plants when compared to salt-stressed non-inoculated plants under salinity stress. Similar improvement of plant growth by symbiotic plant growth-promoting bacteria has been reported with other halophytes such as *Limonium sinense, Salicornia* sp. *Elaeagnus aangustifolia* L., and *Puccinellia tenuiflora* (Qin et al., 2018; Komaresofla et al., 2019; Pan et al., 2020; Bueno and Cordovilla, 2021). Roots are the first “bar of defense” when growing in a saline soil, and root-system indicators are often used to quantify the acquisition capacity of water and nutrients in plants (Brundrett, 1991). Interestingly, *Glutamicibacter* sp. inoculation led to a higher root dry weight compared to non-inoculated stressed plants and alleviated significantly biomass due to salinity, which reflects the promotive effect of *Glutamicibacter* sp. under stress conditions. This also corroborates previous studies highlighting that PGPR colonization positively modulated the root-system architecture and growth under salt stress conditions (Egamberdieva et al., 2017; El-Esawi et al., 2018). PGPR are known to increase root growth of the plant by lowering the ethylene concentration by ACC deaminase in stressing conditions (Qin et al., 2018). The growth improvement recorded in *S. fruticosa* inoculated plants exposed to salt stress may be due to the various PGP characteristics of the inoculated strains. *Glutamicibacter* sp. and *Pseudomonas* sp. genus have been reported for solubilization of phosphorus and production of plant growth regulators (like IAA), ACC, and EPS extracellular (Hidri et al., 2016; Qin et al., 2018). Inoculation with both bacteria increased the shoot: root ratio of *S. fruticosa* plants under NaCl stress, which is consistent with previous findings on the halophyte grass *P. tenuiflora*, where inoculation with *Bacillus subtilis* GB03 strain was found to affect the root: shoot ratio (Bueno and Cordovilla, 2021).

To survive under salt stress conditions, it is essential for plants to maintain lower Na^+^ and Cl^–^ contents in their tissues (Esechie et al., 2002). Thus, controlling Na^+^ homeostasis is critical to maintain normal plant growth during salt stress. In our study, PGPR inoculation decreased Na^+^ content in shoots, strongly suggesting an efficient inhibition of translocation. This could be achieved *via* tissue-specific regulation of HKT1, a plasma membrane Na^+^ uniporter, or by promoting biofilm formation on root surfaces, thus restricting Na^+^ influx into roots (Zhang et al., 2008; Gerhardt et al., 2017). Talaat et al. (2015) suggested that this may be due to the dilution effect associated with the plant growth improvement and the enhanced availability of P in rhizosphere that reduced Na^+^ uptake under saline conditions. Limited Na^+^ influx is thought to be a salt tolerance mechanism that protects the photosynthetic apparatus of *S. fruticosa* from salt damage by decreasing Na^+^ ion translocation to the aerial part (Islam et al., 2016).

Regarding plant nutrition, K^+^ is the pivotal inorganic ion that participates in cellular osmotic adjustment, and absorption of K^+^ improves the water uptake capacity of plants and, consequently, alleviates salt-induced osmotic stress (Tallapragada, 2017). In our study, *Pseudomonas* sp. inoculation enhanced potassium contents in *S. fruticosa* shoots, and this effect may be involved in the maintenance of the turgor pressure and the mitigation of oxidative stress imposed by excessive salinity (Upadhyaya et al., 2011). It is also likely that the increased K^+^ accumulation in salt-stressed plants may be related to the effect of PGPR inoculation on the stability of membranes that facilitates compartmentalization within vacuoles and selective K^+^ uptake. These two elements might be additional factors explaining the mitigation of salt stress imposed by high salinity (Ramoliya et al., 2004). Higher root Ca^2+^ content under salt stress also appears to partly contribute to the improved growth of the investigated species, too. Interestingly, *Pseudomonas sp*. inoculation increased Ca^2+^ in shoots of *S. fruticosa* compared to non-inoculated stressed plants, supporting a possible PGPR inoculation-mediated Ca^2+^ uptake for Na^+^ homeostasis at cellular/tissue level. Thus, we hypothesize that utilization of inorganic ions (potassium and calcium ions) in *Pseudomonas* sp. inoculated plants relieves the physiological drought under salt-induced osmotic stress, strengthens the osmotic adjustment capacity, and allows the allocation of the energy to be used for growth to a certain extent.

Soil salinity increases cellular levels of reactive oxygen species ROS such as superoxide radicals (O_2_^⋅⁣–^) and H_2_O_2_ that lead to lipid peroxidation of membranes (Gill and Tuteja, 2010) and increases the content of the biomarker, MDA (Das and Roychoudhury, 2014), and the relative electric conductivity (Noctor et al., 2014). In this study, *S. fruticosa* inoculated plants with both bacterial strains exhibited lower MDA contents than non-inoculated plants under saline condition, indicating a lower accumulation of ROS and membrane damage. The reduction in MDA content suggests that inoculation better protects the plants from the imposed oxidative stress caused by NaCl. Antioxidant enzyme activities upon *Glutamicibacter* sp. inoculation were higher than those of non-inoculated salt-stressed *S. fruticosa* plants. This could be explained by the fact that *Glutamicibacter* sp. inoculation enhanced the synthesis of these enzymes. Overall, higher enzyme activities in plants inoculated with *Glutamicibacter* sp. were associated with lower amount of lipid peroxidation, indicating a lower oxidative burden and less membrane destruction in these plants. Our results are consistent with previous studies of the coastal halophyte *L. sinense* and *Salicornia* sp. inoculated with *Glutamicibacter halophytocola* KLBMP 5180 and *Staphylococcus* sp. R11, respectively, and treated with salt (Qin et al., 2018; Komaresofla et al., 2019). Liu et al. (2017) showed that *Bacillus amyloliquefaciens* FZB42 induces salt tolerance in *Arabidopsis thaliana* through enhancing expression of genes linked to ROS-scavenging enzymes. The ameliorative effect on MDA accumulation in plants inoculated with *Pseudomonas* sp. maybe also related to the higher accumulation of nitrogen-containing compounds such as proline (Hidri et al., 2016), which is involved in the stabilization of sub-cellular structures (membrane and proteins), scavenge free radicals, and buffer cellular redox potential under stress conditions (Kishor et al., 2005).

Microbial inoculation not only enhances plant biomass (Puente et al., 2013) but also improves soil microbial activities (Bharti et al., 2015). Plant growth is inseparable from nutrient uptake by plants. According to Caldwell (2005), soil enzyme activities reflect the soil community metabolic demands and available nutrients, since changes in soil nutrients are closely related to soil enzyme activities, which are of major significance for plant nutrition processes (Tirry et al., 2021).

As key indicator of ecosystem health and sustainability, soil enzyme activity plays a potentially crucial role in soil biochemical reactions (Cao et al., 2014; Sipahutar et al., 2018). In this study, three hydrolases (urease, dehydrogenases, and ß glucosidase) involved in the geochemical cycling of processes of N, H, and C were considered. Assaying dehydrogenase, an enzyme which typically exists in every viable microbial cells (Singh and Kumar, 2008), provides a reliable estimate of the overall metabolic activity of microorganisms in soils (Cao et al., 2014). We confirmed that dehydrogenase activity of inoculated soil was higher than non-inoculated soil under saline stress. Previous studies have documented that dehydrogenase activity might increase following bacterial inoculation and suggested that the soil microbial biomass is correlated with this activity because it is an intracellular enzyme that is implicated in microbial oxidoreductase metabolism (Taylor, 2002; Cao et al., 2014).

Inoculation with *Pseudomonas* sp. increased significantly both urease and β-glucosidase activities of the rhizosphere soil under saline condition. Urease is one of the most commonly assayed soil enzymes, because it greatly influences the hydrolysis of urea to ammonia (Caldwell, 2005; Rodríguez-Caballero et al., 2017). Soil β-glucosidase is also considered as a useful soil quality indicator related to C cycling (Stott et al., 2010). Data inferred from the present study indicate that the activity of soil enzymes (dehydrogenase, urease, and β-glucosidase) was severely inhibited in saline soils inoculated with *Glutamicibacter* sp. as compared to non-saline soil. This might be due to the “salting-out” effect on enzyme proteins through the disruption of the tertiary protein structure and/or to the decrease in abundance and the activity of soil microorganisms (Rietz and Haynes, 2003; Zhang et al., 2011; Folli-Pereira et al., 2013; Islam et al., 2016). These findings further confirm the assumption that the effects of microbial inoculation on soil enzyme activities are enzyme-specific and strongly influenced by soil salinity (Cao et al., 2014). Hence, the effect of the inoculation with the selected bacteria in alleviating NaCl stress on soil enzyme activities is important to consider in the biochemical response of the plant–soil system under saline conditions.

The microbial production of plant growth regulators such as IAA is an important parameter regarding soil fertility, and it is frequently cited as a potent mechanism of PGPR to increase plant growth (Glick, 2012). Our results indicated that both bacterial strains we investigated increased the level of IAA in the rhizospheric soil under salt stress conditions. IAA regulates several aspects of plant growth and development by controlling critical biological processes, such as lateral root initiation, cell extension, cell division, and increase root surface area that helps the plants maintain sufficient soil nutrient uptake (Zhao, 2010). IAAs promote plant growth not only under normal conditions but also under different stress conditions. However, we cannot ignore the fact that the higher development of the root system induced by IAA enhances the ability of plants to take up water and also results in high internal Na^+^ and Cl^–^ concentrations that can reach toxic levels. In the case of inoculated plants, decreased Na^+^ and Cl^–^ accumulation could be explained by the excretion of specific exuded PGPR compounds such as EPS, which provides more nutrients and excludes toxic ions (Na^+^ and Cl^–^) from the rhizosphere, thus allowing the host to develop thicker roots and greater uptake and helping alleviate salt stress in plants (Pan et al., 2020). Bacterial EPS has the potential to bind cations including Na^+^, thus making it inaccessible to plants under saline conditions (Mukhtar et al., 2019). According to Rhaman et al. (2021), seed priming with IAAs at 50 ppm enhanced tolerance to drought stress by improving antioxidant enzyme activities such as POD, CAT, and SOD. In our study, positive correlations were found under *Glutamicibacter* sp. inoculation (S + Glu) for IAA content in the soil and antioxidant enzyme activities (CAT, APX, and GR).

## Conclusion

*Glutamicibacter* sp. and *B. subtilis* strains might play an important ecological function, conferring increased growth and salt tolerance of *S. fruticosa* on host plants likely through its synergetic effect on several physiological and biochemical mechanisms that improve the plant response. Plant inoculation with *Pseudomonas* sp. increased plant antioxidative capacity and decreased oxidative stress severity, whereas inoculation with *Glutamicibacter* sp. modulated the soil enzymatic activities under high salinity. *Glutamicibacter* sp. and *B. subtilis* strains are promising candidates for the phytoremediation and rehabilitation of saline soils in future. In addition, the domestication of wild halophytes as crops with bacterial inoculation appears to be a viable method to promote agriculture in highly saline habitats.

## Data Availability Statement

The original contributions presented in the study are included in the article/supplementary material; further inquiries can be directed to the corresponding author.

## Author Contributions

RH performed the experiments, analyzed data, and wrote the manuscript. OM-BM, CA, and RA conceived and designed the study. WZ, AD, and HM contributed in data analysis. AD and WZ revised the manuscript. All authors contributed to the article and approved the submitted version.

## Conflict of Interest

The authors declare that the research was conducted in the absence of any commercial or financial relationships that could be construed as a potential conflict of interest.

## Publisher’s Note

All claims expressed in this article are solely those of the authors and do not necessarily represent those of their affiliated organizations, or those of the publisher, the editors and the reviewers. Any product that may be evaluated in this article, or claim that may be made by its manufacturer, is not guaranteed or endorsed by the publisher.
